# Advancing social determinants of health research and practice: Data, tools, and implementation

**DOI:** 10.1017/cts.2024.657

**Published:** 2025-03-24

**Authors:** Chenyu Li, Jingchuan Guo, Jiang Bian, Michael J. Becich

**Affiliations:** 1 Department of Biomedical Informatics, School of Medicine, University of Pittsburgh, Pittsburgh, PA, USA; 2 Department of Pharmaceutical Outcomes and Policy, College of Pharmacy, University of Florida, Gainesville, FL, USA; 3 Department of Health Outcomes and Biomedical Informatics, College of Medicine, University of Florida, Gainesville, FL, USA

Social determinants of health (SDoH) gained increasing attention in recent years, recognizing their profound impact on health outcomes and equity. It is important to distinguish SDoH from related concepts. SDoH refers to the conditions in which people are born, grow, live, work, and age, road societal and environmental conditions affecting population health; social risks stand for individual-level adverse social conditions associated with poor health; and health-related social needs refers to immediate, individual-level social and economic factors that patients identify as key drivers of their health [1].

Advances in artificial intelligence (AI) and the proliferation of real-world data (RWD), such as electronic health records (EHRs) and administrative claims data, have created unique opportunities to develop innovative approaches that address these social factors, ultimately improving health outcomes and equity. The diverse range of studies presented in this special issue spans an impressive array of topics, highlighting the multifaceted nature of SDoH research at the intersection of clinical and translational science and informatics. The accepted publications range from the development of innovative SDoH data infrastructure leveraging RWD to the creation and implementation of cutting-edge tools employing advanced artificial intelligence and machine learning (AI/ML) techniques, to the real-world application of informatics tools designed to enhance community outreach and promote health equity.

These contributions push the boundaries of what’s possible in research and practice that aim to address SDoH – often the root causes of health disparities and health inequity, using clinical translational science as a vehicle and informatics as a key tool [2,3]. For example, these studies showcase rigorous assessments of best practices in integrating SDoH, alongside novel methodological approaches and creative strategies to improve diversity in clinical research enrollment by addressing social factors. It’s worth noting that the importance of addressing SDoH is increasingly recognized at the policy level. In 2024, the Joint Commission and the Centers for Medicare and Medicaid Services will begin requiring the collection of SDoH data, underscoring the growing emphasis on these factors in healthcare delivery and policy [4]. Additionally, the White House has recently released a comprehensive SDoH Playbook [5], providing guidance on addressing these critical social factors across various sectors.

Together, these studies and policy developments paint a comprehensive picture of the current state of SDoH research and practice while also illuminating the path forward for developing not only effective but also efficient learning health systems and beyond the learning health communities that consider the social needs of the populations they serve. Among the total of 36 papers of this special issue, we classified into four distinct categories: data infrastructure (*n*= 7), tool development (*n*= 8), tool implementation (*n*= 10), and community outreach (*n*= 9), along with two review papers. In this editorial, we focus on the first three categories, aiming to summarize the current state and progress in leveraging RWD and AI to address SDoH in clinical and translational research, as well as discuss future opportunities. A separate editorial will focus on articles related to community outreach [6].

## SDoH data infrastructure

Building robust data infrastructure and data ecosystems is a critical first step for SDoH research and practice. While SDoH factors are often categorized into two main categories – contextual SDoH (i.e., neighborhood-level or place-based factors) and individual-level social risks and needs – the integration of this information with existing healthcare data presents both challenges and opportunities [7,8].

Contrary to the initial oversimplification, many healthcare systems have made significant strides in linking EHR data with area-level SDoH information. However, the seamless integration of this data into clinical care workflows and decision-making processes remains an area for improvement. Similarly, while an increasing number of clinics and hospitals are now screening patients for social risks and needs, the comprehensive integration and utilization of this information across the healthcare system is still evolving.

The current special issue accepted a total of 7 papers [9–15] dedicated to SDoH data, with 5 specifically focused on data infrastructure building and tool development. These contributions represent significant advancements in efforts to build SDoH data infrastructure, offering new opportunities to enhance our ability to address health disparities via SDoH research and practice. Craven et al. [16] introduced their collaborative efforts on building a SDoH data infrastructure across four Texas Clinical and Translational Science Award (CTSA) institutions, promoting SDoH data standardization, harmonization, and integration into EHRs. Similarly, Abdallah et al. [14] introduced their procedure for integrating social and environmental determinants (i.e., contextual SDoH) by geocoding patients’ residential addresses to geo-enrich an EHR data warehouse in a secure and compliant manner.

Three papers report on their SDoH data platform products. Tilmon et al. [9] presented the Sociome Data Commons (SDC), a cloud-based repository of harmonized, geospatial sociome data sets. This platform, designed to work alongside clinical data, predicts a variety of health outcome by characterizing local social, environmental, behavioral, psychological, and economic exposures. The SDC adheres to principles of governance, sustainability, inclusiveness, disaggregation, and multidisciplinary management as well as the Findable, Accessible, Interoperable, and Reusable (FAIR) principles for its data products. As a use case, they investigated social factors associated with asthma exacerbations in children residing on the South Side of Chicago, using machine learning and six SDC datasets. Holt et al. [10] introduced RocHealthData.org, a web-based tool providing access to thousands of publicly available national datasets. The platform already has 1033 registered users, with 28.2% from the community. Adams et al. [11] described the development and evaluation of “Health Equity Explorer (H2E),” an open-source, scalable informatics tool designed to support dynamic and interactive explorations of diverse health drivers compatible with RWD structured in the Observational Medical Outcomes Partnership Common Data Model.

Two additional papers by Bensken et al. [17] and Telzak et al. [12] are focused on examining the correlation between contextual-level SDoH and individual-level SDoH. Although both studies showed suboptimal associations, the study by Telzak et al. [12] suggested the strength of these associations and predictive values were better in areas with high SDoH risk compared to areas with low SDoH risk.

These contributions represent significant advancements in efforts that build SDoH data infrastructure, offering new opportunities to enhance our ability to address health disparities via SDoH research and practice.

## SDoH tool development

Development of tools plays a crucial role in addressing SDoH by providing innovative solutions to identify, measure, and analyze the complex SDoH factors influencing health outcomes. The papers featured in this special issue highlight a range of notable tools and methodologies designed to tackle various aspects of SDoH [17–24]. For example, German et al. [21] presented an interactive visualization tool that integrates clinical, sociodemographic, and environmental data to enhance understanding of health disparities in diabetes care and outcomes. Harris et al. [20] introduced a Natural Language Processing (NLP) tool for identifying housing instability and supporting clinical trials related to housing issues. Huang et al. [23] developed advanced NLP models for identifying incarceration history, an important SDoH factor, from unstructured clinical notes, opening new avenues for targeted interventions and research. Resnick et al. [18] developed a terminological representation of the Assessing Circumstances & Offering Resources for Needs SDoH survey, addressing a gap in representing SDoH with standardized terminologies and enabling its use with NLP and clinical decision support systems. Bensken et al. [17] assessed the predictive ability of area-level SDoH measures for individual-level social risks and explored biases in social risk screening. Espinoza et al. [19] proposed a Social and Environmental Determinants of Health (SEDoH) Informatics Maturity Model to help organizations assess their current level of SEDoH informatics maturity and identify areas for improvement. Ngai et al. [22] developed a method to measure concordance and discordance between individual characteristics and corresponding neighborhood-level SDoH, enhancing our understanding of their interplay and impact on health. These tools and methodologies have the potential to advance clinical and translational research by enabling researchers to better understand the complex interplay between SDoH and health outcomes, ultimately informing the development of targeted interventions to address health disparities.

## SDoH associated tool implementation and knowledge generation

The landscape of SDoH research and practice is undergoing a transformative shift, driven by innovative tools and technologies that enhance our understanding and ability to address health disparities. The papers featured in this special issue showcase notable implementations that are pushing the boundaries of SDoH informatics and intervention strategies [25–35]. From comprehensive organizational assessment frameworks [25,31] to cutting-edge NLP techniques [23], these advancements represent a significant leap forward in our ability to capture, analyze, and utilize SDoH data effectively.

Particularly noteworthy is the trend toward leveraging technology to overcome longstanding barriers in SDoH research and intervention. The success of remote enrollment protocols in increasing participation among underrepresented groups in clinical trials [36] is a prime example of how digital approaches can enhance inclusivity and representation. These implementations, along with efforts to standardize SDoH terminology [35] and assess organizational readiness [19], collectively point to a future where SDoH data is not just collected but also seamlessly integrated into clinical decision-making and population health strategies. As we move forward, it is imperative that healthcare systems and researchers embrace these tools and technologies, recognizing their potential to drive more equitable health outcomes and inform evidence-based policies to address the root causes of health disparities.

## Summary and looking forward

**Figure 1.**
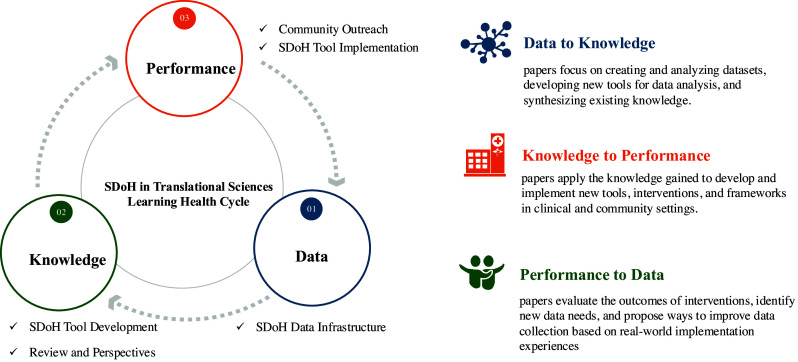
Social determinants of health-driven translational sciences: a learning health cycle from data to action.

Collectively, this body of work underscores the importance of leveraging cutting-edge informatics methodologies for building SDoH data infrastructure, as well as developing and implementing tools to address SDoH and reduce health disparities. Figure 1 provides a comprehensive overview of the papers related to informatics and data infrastructure that were accepted for the SDoH special issue in the Journal of Clinical and Translational Sciences. Utilizing a Learning Health Systems framework [37], Figure 1 illustrates how these contributions collectively advance the integration of SDoH into clinical and translational sciences. The inner circle depicts a continuous cycle of Data, Knowledge, and Performance, which encompasses the broad topics covered in the special issue.

As we reflect on the diverse range of studies presented in this special issue, several key themes emerge that will shape the future of SDoH research and practice. The importance of robust data infrastructure and standardization is evident across multiple studies, providing a solid foundation for more comprehensive analyses of health disparities. The development of sophisticated tools and methodologies, particularly in NLP, has also emerged as a significant trend. These studies not only highlight technical solutions but also raise important questions about data privacy and the ethical use of information.

The focus on implementation and real-world assessment underscores the importance of bridging the gap between theoretical models and practical applications. The studies presenting “best practices” and use cases offer a roadmap for institutions and communities looking to enhance their SDoH-related initiatives. Interestingly, while our initial call emphasized AI and machine learning methods broadly, the contributions we received primarily focused on NLP applications, suggesting that text-based data sources may be a particularly rich resource for understanding and addressing SDoH.

When examining the connections between seemingly disparate manuscripts, we see a cohesive story emerging. For instance, the work on data infrastructure by Craven et al. [16] and Abdallah et al. [14] provides the foundation upon which tools like the Health Equity Explorer by Adams et al. can be built [11]. These tools, in turn, inform implementation strategies like those described by Fiori et al. [25] and LeLaurin et al. [26]. This progression from data to tools to implementation illustrates the interconnected nature of SDoH research and the importance of a comprehensive approach.

Moreover, the studies by Bensken et al. [17] and Telzak et al. [12] highlight the complexity of these social factors and the need for nuanced approaches in both research and clinical practice. This complexity is further explored in the implementation studies, such as Miller et al.’s work in a free clinic setting [27], demonstrating how SDoH research can directly impact patient care in diverse healthcare environments. Li et al. conducted a scoping review of approaches for integrating SDoH data into EHR systems, offering a comprehensive overview of current practices and future possibilities for leveraging this data in clinical settings [24].

The field of SDoH research is continually evolving, and as we consider its future, it’s imperative to acknowledge the critical role of community engagement, as highlighted by Cottler [6]. Community engagement provides insights beyond what can be gleaned from medical records alone, ensuring that SDoH initiatives address the actual needs and priorities of the entire community, not just those represented in medical databases.

As we conclude this special issue, we are filled with a sense of optimism, tempered by the recognition of the significant work that still lies ahead. We hope this collection serves as a catalyst for ongoing innovation, collaboration, and progress in the vital field of SDoH. The integration of advanced informatics methodologies, robust data infrastructure, and community-engaged research holds the promise of creating truly transformative solutions for addressing health disparities.

We urge researchers, clinicians, informaticians, policymakers, and community leaders to embrace the challenges and opportunities that come with SDoH research. Let us commit to fostering interdisciplinary collaboration, prioritizing community engagement, and advocating for policies that address the root causes of health inequities. Together, we can build a more equitable, effective, and compassionate healthcare system that truly serves all members of society.
